# PCSK9 inhibitor improves cardiac function and reduces infarct size in rats with ischaemia/reperfusion injury: Benefits beyond lipid‐lowering effects

**DOI:** 10.1111/jcmm.14586

**Published:** 2019-09-26

**Authors:** Siripong Palee, Christian M. McSweeney, Chayodom Maneechote, Dalila M. Moisescu, Thidarat Jaiwongkam, Sasiwan Kerdphoo, Siriporn C. Chattipakorn, Nipon Chattipakorn

**Affiliations:** ^1^ Cardiac Electrophysiology Research and Training Center, Faculty of Medicine Chiang Mai University Chiang Mai Thailand; ^2^ Center of Excellence in Cardiac Electrophysiology Research Chiang Mai University Chiang Mai Thailand; ^3^ School of Biological Sciences, Faculty of Biology, Medicine and Health The University of Manchester Manchester England; ^4^ Cardiac Electrophysiology Unit, Department of Physiology, Faculty of Medicine Chiang Mai University Chiang Mai Thailand; ^5^ Department of Oral Biology and Diagnostic Sciences, Faculty of Dentistry Chiang Mai University Chiang Mai Thailand

**Keywords:** heart, ischaemia, mitochondria, PCSK9 inhibitor, reperfusion injury

## Abstract

During acute cardiac ischaemia/reperfusion (I/R), an increased plasma proprotein convertase subtilisin/kexin 9 (PCSK9) level instigates inflammatory and oxidative processes within ventricular myocytes, resulting in cardiac dysfunction. Therefore, PCSK9 inhibitor (PCSK9i) might exert cardioprotection against I/R injury. However, the effects of PCSK9i on the heart during I/R injury have not been investigated. The effects of PCSK9i given at different time‐points during I/R injury on left ventricular (LV) function were investigated. Male Wistar rats were subjected to cardiac I/R injury and divided into 3 treatment groups (n = 10/group): pre‐ischaemia, during ischaemia and upon onset of reperfusion. The treatment groups received PCSK9i (Pep2‐8, 10 μg/kg) intravenously. A control group (n = 10) received saline solution. During the I/R protocol, arrhythmia scores and LV function were determined. Then, the infarct size, mitochondrial function, mitochondrial dynamics and level of apoptosis were determined. PCSK9i given prior to ischaemia exerted cardioprotection through protection of cardiac mitochondrial function, decreased infarct size and improved LV function, compared with control. PCSK9i administered during ischaemia and upon the onset of reperfusion did not provide any of those benefits. PCSK9i administered before ischaemia exerts cardioprotection, as demonstrated by the attenuation of infarct size and cardiac arrhythmia during cardiac I/R injury. The attenuation is associated with improved mitochondrial function and connexin43 phosphorylation, leading to improved LV function.

## 1 INTRODUCTION

Cardiovascular disease (CVD) currently represents the highest incidence of mortality worldwide.^1^ Hyperlipidemia associated with both genetic pre‐disposition and obesity represents a significant risk factor for CVD, and thus, lipid‐lowering drugs are an important avenue for disease prevention. Currently, therapeutics used to lower serum lipid levels include statins, ezetimibe and fenofibrates.^2^ However, many patients do not reach an adequate reduction in LDL‐C. Thus, alternatives such as proprotein convertase subtilisin‐kexin type 9 (PCSK9) inhibitors are being sought to derive a new era for cardioprotective medicine.^3^ Previous research, both pre‐clinical and clinical, has highlighted the success of PCSK9 inhibitors in the reduction in serum LDL‐C and atheroprotection.^4^

PCSK9 is a 692 amino acid serine protease, expressed predominantly in hepatocytes.^5^ Physiological lipid homeostasis involves LDL‐C transportation to LDL receptors (LDLR) found on hepatocytes involved in circulatory clearance. PCSK9 can bind to the LDLR and influence receptor degradation, reducing the overall density of LDLR found on the surface of hepatocytes.^6^ Consequently, lipid clearance from circulation is limited and the serum lipid concentration is raised. The inhibition of PCSK9, therefore, acts to attenuate PCSK9‐led LDLR degradation, increase lipid clearance and lower serum LDL‐C.^7^ Since hyperlipidemia has been shown to aggravate myocardial ischaemia‐reperfusion (I/R) injury and attenuate the cardioprotective effects of pre‐conditioning, a lipid‐lowering drug might mitigate I/R injury.^8^

Under conditions of hypoxia, such as those associated with acute myocardial infarction (AMI), cardiac tissue is not adequately perfused. Reperfusion of tissue is essential to regain function. However, this in itself can induce significant adverse effects on the myocardium, including induction of mitochondrial stress.^9^ Reactive oxygen species (ROS), such as free radicals and oxides, can exert effects on the mitochondrial permeability transition pores (mPTP) leading to mitochondrial stress, demonstrated by mitochondrial depolarization and swelling.^10^ This in turn increases cytochrome *c* release, modifies Bcl‐2/Bax dynamics and ultimately results in the induction of caspase 3‐mediated cardiomyocyte apoptosis.^11^ This is known to increase infarct size during cardiac I/R.^12^ It has been shown that infarct size post‐I/R is inversely correlated with cardiac function and is associated with poor patient outcome.^13^ Moreover, a previous study demonstrated that ROS levels were correlated with PCSK9 expression.^14^ Thus, the inhibition of PCSK9 may potentially act to limit mitochondrial stress during I/R injury and ultimately result in improved cardiac function.

Although the cardioprotective effects of PCSK9 inhibitor had been reported in mice, this was done in chronic myocardial infarction model. Moreover, findings from that study 15 had a limitation for its clinical application to acute myocardial infarction cases since PCSK9 inhibitor was given only prior to myocardial ischaemia. In our study, acute administration of PCSK9 inhibitor was done in an acute myocardial ischaemia/reperfusion injury model in rats. To emphasize the importance of our study design, the comparative effects of PCSK9 inhibitor given at different time‐points were reported here, which had never been reported previously. Therefore, in this study we sought to investigate the effects of PCSK9 inhibitor administration in the rodent cardiac I/R model on myocardial infarct size, arrhythmias, left ventricular (LV) function and mitochondrial function and dynamics. To investigate these effects, PCSK9 inhibitor was administered at several time periods associated with coronary occlusion (pre‐ischaemia, during ischaemia and at the onset of reperfusion). We hypothesized that PCSK9 inhibitor exerts cardioprotection, as demonstrated by attenuated LV dysfunction during cardiac I/R injury.

## 2 METHODS

### 2.1 Animal preparation

All procedures followed during this investigation conform to the ARRIVE and the United States NIH guidelines (Guide for the care and use of laboratory animals), and all animal experiments in this investigation were approved by the Faculty of Medicine, Chiang Mai University Institutional Animal Care and Use Committee, and all experimental were performed in Cardiac Electrophysiology Research and Training Center, Faculty of Medicine, Chiang Mai University, Thailand.

Male Wistar rats weighing 300‐350 g (8 weeks old) were obtained from the National Laboratory Animal Centre, Mahidol University, Bangkok, Thailand. The rats were housed under constant environmental conditions (temperature at 22‐25°C and a 12‐hour light/dark cycle), with standard pelted rat diet and water ad libitum. All animals were maintained in environmentally controlled conditions (25 ± 0.5°C, 12‐hour light/12‐hour dark cycle) and fed with normal rat chow and water ad libitum for 1 week to allow for acclimatization before study.

### 2.2 Surgical preparation of myocardial I/R model in rats

Rats were anesthetized with an intramuscular injection of Zoletil (50 mg/kg) and xylazine (0.15 mg/kg). Rats were mechanically ventilated with room air after a tracheotomy was performed along the ventral midline. Electrocardiogram (ECG) was recorded throughout the experiment. A left side thoracotomy was performed, and the left anterior descending coronary artery (LAD) was ligated using a surgical suture, occluding 2 mm distal to the origin. The LAD was occluded for 30 minutes prior to reperfusion for a further 120 minutes. Blanching of the myocardium and ST elevation on the ECG confirmed cardiac ischaemia.

### 2.3 Experimental design

**Figure 1:**
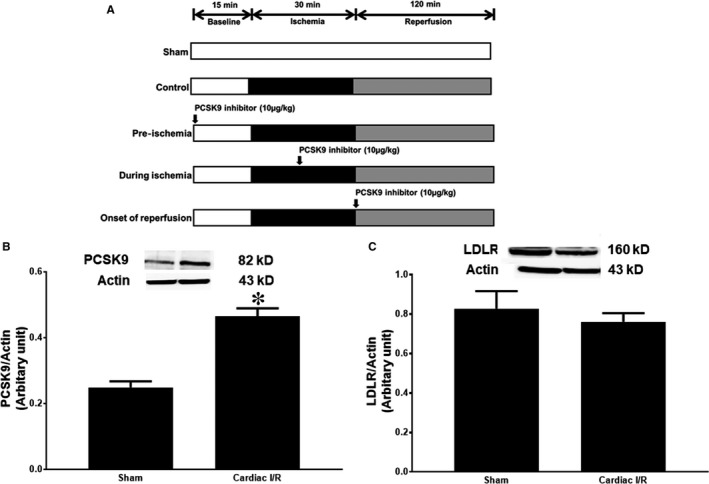
(A) Study protocol of the effects of PCSK9 inhibitor given at different time‐points (pre‐ischaemia, during ischaemia and at onset of reperfusion) during acute I/R injury in lean rats, (B) The effects of cardiac I/R injury on PCSK9 expression and (C) LDLR expression. *P < .05 vs sham

Rats were randomly divided into four subgroups to receive different treatments via intravenous injection (Figure 1A). (1) Pre‐treated group: received the PCSK9 inhibitor 15 minutes before ischaemia; (2) Ischaemia group: received the PCSK9 inhibitor 15 minutes after the onset of ischaemia; (3) Reperfusion group: received the PCSK9 inhibitor at the onset of reperfusion and (4) Control group: received normal saline. PCSK9 inhibitor (Pep2‐8 trifluoroacetate, 10 µg/kg, Sigma‐Aldrich) or the saline was administered via intravenous injection through the femoral vein. During the I/R protocol, the LV function was recorded using a pressure‐volume (P‐V) loop recording system (Transonic Scisense Inc). A surface electrocardiogram (ECG) was used to record and determine an arrhythmia score and the mortality rate. At the end of the I/R protocol, the heart was rapidly removed, following decapitation under deep anaesthesia, for infarct size measurement and cardiac tissue studies.

### 2.4 Arrhythmia determination

Power Lab 4/25T (AD Instruments, Inc, CO, USA) was used to record the ECG Lead II with a sampling rate of 100 times/sec. The ECG was recorded via needle electrodes inserted subcutaneously into the positions of lead II ECG. Arrhythmia score and time to the first ventricular tachycardia/ventricular fibrillation (VT/VF) onset were determined. The Lambeth Convention was used to measure the occurrence of arrhythmia, and the arrhythmia score was characterized in accordance with the Curtis and Walker criteria.^16^

### 2.5 Left ventricular function measurement

A pressure‐volume (PV) catheter (Transonic Scisense Incl.) was inserted into the exposed right common carotid artery during I/R and directed into the left ventricle. Left ventricular function parameters such as stroke volume (SV), end‐systolic pressure (ESP), end‐diastolic pressure (EDP) and maximum + dP/dt were measured using analytical software (Labscribe).^17^

### 2.6 Infarct size measurement

At the end of the study protocol, the rat was decapitated and the heart was rapidly removed. Then, the LAD was occluded again at the same occlusion site used during I/R. The heart was perfused with 1 mL Evan's blue dye via the aorta to determine the LV area at risk (AAR). The heart was frozen at −20°C overnight and then sectioned horizontally from the apex to the occlusion site into 7‐9 slices. Then, 2, 3, 5‐triphenyltetrazolium chloride (TTC) was added to each slice for 15 minutes, and the area with viable tissues seen in red was measured. The infarct size was determined from the area that was not stained with Evan's blue and TTC. The infarct size and the AAR were determined using the image tool software version 3.0 and were calculated according to the Reiss et al formula.^18^

### 2.7 Isolated cardiac mitochondria studies

At the end of the I/R protocol, the heart was removed, and cardiac mitochondria were isolated from the ischaemic and non‐ischaemic areas of the ventricles as described previously.^19^ The protein concentration was determined according to a Bicinchoninic Acid (BCA) assay. The isolated cardiac mitochondria were used to determine mitochondrial function including mitochondrial reactive oxygen species (ROS) levels, mitochondrial membrane potential and mitochondrial swelling. Cardiac mitochondrial function was determined as has been described previously.^20^ In brief, the mitochondrial ROS level was measured using a dichlorohydro‐fluorescein diacetate dye (DCFDA). DCFDA is oxidized in the presence of H_2_O_2_ to dichlorofluorescein (DCF). The fluorescence intensity of the DCF was measured using a fluorescent microplate reader (excitation at 485 nm and emission at 530 nm). The ROS levels were expressed as arbitrary units of fluorescence intensity of DCF.^20^

The change in mitochondrial membrane potential was measured using 5,5′,6,6′‐tetrachloro‐1,1′,3,3′‐tetraethylbenzimidazolcarbocyanine iodide dye (JC‐1). JC‐1 is a ratiometric dye which is internalized as a monomer dye (green fluorescence, excitation at 485 nm and emission at 530 nm) and is concentrated by respiring mitochondria with a negative inner membrane potential into a J‐aggregate dye (red fluorescence, excitation at 485 nm and emission at 590 nm). The intensity of fluorescence was determined using a fluorescent microplate reader. The change in mitochondrial membrane potential was calculated from the ratio of red to green fluorescence. In this study, mitochondrial depolarization is indicated by a decrease in the red/green fluorescence intensity ratio.^20^

The isolated mitochondrial suspension was used to measure the change in the absorbance of the mitochondrial suspension detected at 540 nm by using a microplate reader. Mitochondrial swelling was indicated by a decrease in the absorbance of the mitochondrial suspension. In addition, transmission electron microscopy (TEM) was used to determine the morphology of isolated cardiac mitochondria.^20^

### 2.8 Western blot analysis

Cardiac tissue samples were divided into non‐ischaemic (remote) and ischaemic areas and added to lysis buffer. Protein concentration was determined using a Bio‐Rad protein assay kit (Bio‐Rad Laboratories), the proteins being added to the loading buffer.^19^ The proteins were then transferred to nitrocellulose membranes with transfer buffer. Skimmed milk (5% solution) was used to block the membranes for 1 hour at room temperature. The nitrocellulose membranes were exposed overnight to anti‐LDLR, anti‐PCSK9, anti‐Bax, anti‐Bcl2, anti‐caspase 3, anti‐connexin43 (Cx43), anti‐connexin43 phosphorylated at Ser368, anti‐Drp1, anti‐Mfn2 and anti‐VDAC (Cell Signalling technology, Danvers, MA, USA). Subsequently, the membranes were washed and incubated with horseradish peroxidase conjugated with anti‐rabbit IgG (Cell Signaling Technology). Finally, the bands were detected and were used to determine protein expression.^19^

### 2.9 Data analysis

The experimental procedures or treatment and data analyses were carried out with randomization and blinding. Data are presented as mean ± SEM. Values are analysed by two‐way ANOVA, with post hoc Fisher's test to examine the differences between groups. A *P* value < .05 was considered statistically significant.

## 3 RESULTS

### 3.1 Cardiac I/R injury increased PCSK9 expression but did not alter LDLR expression in the heart

Cardiac I/R injury increased PCSK9 expression in cardiac tissues, compared with the sham‐operated group (Figure 1B). However, we found that there were no differences in LDLR expression between groups (Figure 1C).

### 3.2 PCSK9 inhibitor decreased cardiac infarct size during I/R

**Figure 2:**
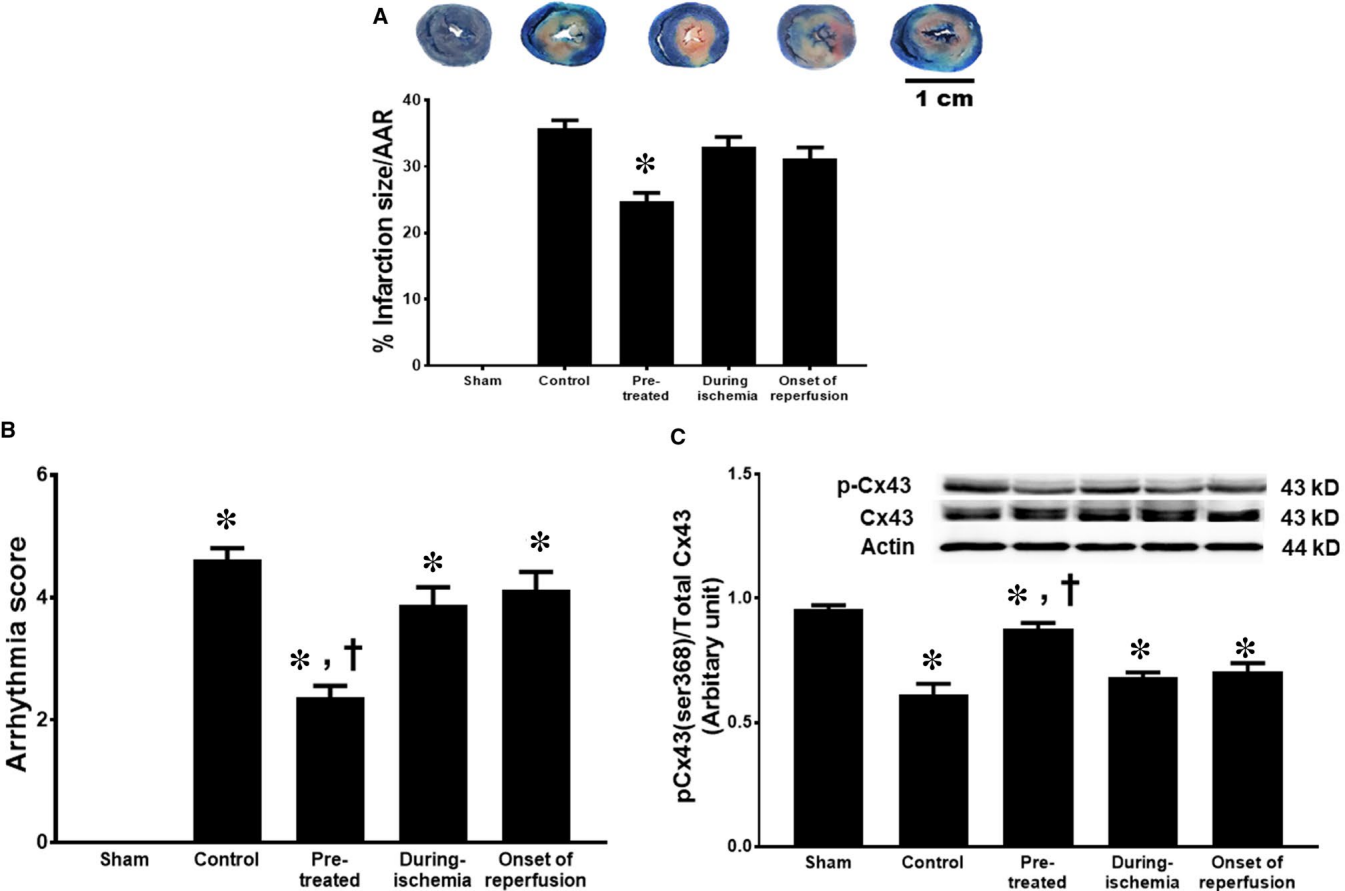
The effect of PCSK9 inhibitor on myocardial infarct size and arrhythmias given at different time‐points (pre‐treatment, during ischaemia and at onset of reperfusion) during cardiac I/R. (A) myocardial infarct size, (B) arrhythmia score and (C) pCx43 (ser368)/total Cx43. AAR: area at risk, pCx43(ser368): phosphorylation connexin43 at serine‐368. *P < .05 vs sham, †*P* < .05 vs control

In this study, the AAR in the control group [45 ± 3%] was not different from that in any groups treated with PCSK9 inhibitor (pre‐treated group [42 ± 1%], during ischaemia [45 ± 1%] and onset of reperfusion [44 ± 2%]). However, only the pre‐treated group had significantly reduced myocardial infarct size, compared with the control group (Figure 2A).

### 3.3 PCSK9 inhibitor treatment was associated with attenuated arrhythmia

The arrhythmia score in the PCSK9 inhibitor pre‐treated group was significantly decreased when compared to the control group (Figure 2B). However, administration of the PCSK9 inhibitor during ischaemia and at the onset of reperfusion did not reduce the arrhythmia score (Figure 2B). Furthermore, the expression of phosphorylated connexin43 at serine 368 (pCx43) in cardiomyocyte tissue increased in the pre‐treated group, compared with the control group (Figure 2C). PCSK9 inhibitor treatment during ischaemia or at the onset of reperfusion had no effect on pCx43 expression, when compared to the control group.

### 3.4 PCSK9 inhibitor pre‐treatment in association with cardiac I/R improved mitochondrial function

**Figure 3:**
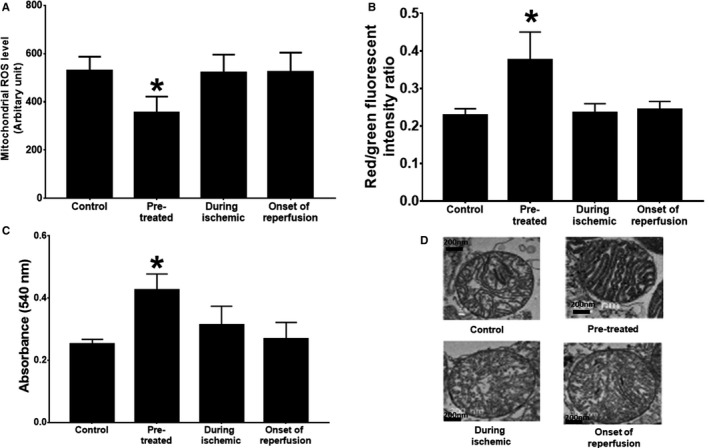
The effect of PCSK9 inhibitor on cardiac mitochondrial function given at different time‐points (pre‐treatment, during ischaemia and at onset of reperfusion) during cardiac I/R. (A) Mitochondrial ROS production; (B) mitochondrial membrane potential; (C) mitochondrial swelling; and (D) TEM representative images of cardiac mitochondria. ROS: reactive oxygen species; TEM: transmission electron microscopy. *P < .05 vs control group

When given prophylactically (pre‐treated group), PCSK9 inhibitor significantly decreased mitochondrial reactive oxygen species (ROS), compared with the control group (Figure 3A). PCSK9 pre‐treatment likewise resulted in a significant improvement on other mitochondrial function parameters, including decreased mitochondrial swelling and mitochondrial depolarization (Figure 3B,C). However, administration of the PCSK9 inhibitor during ischaemia and at the onset of reperfusion did not show significant improvement in any of the mitochondrial function parameters (Figure 3A‐C). These observations showed a correlation with the TEM mitochondrial morphology study, demonstrating mitochondrial swelling was reduced in only the pre‐treated group when compared to other groups (Figure 3D).

### 3.5 PCSK9 inhibitor pre‐treatment reduced apoptosis‐associated protein expression

**Figure 4:**
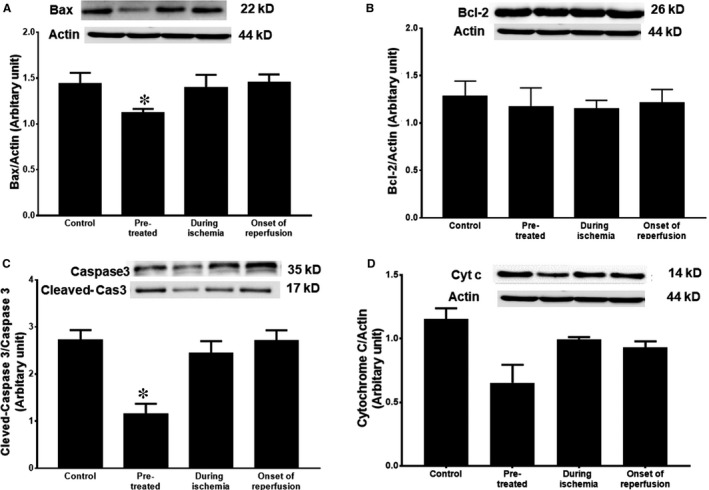
The effect of PCSK9 inhibitor on cardiac apoptosis given at different time‐points (pre‐treatment, during ischaemia and at onset of reperfusion) during cardiac I/R. (A) Bax, (B) Bcl‐2, (C) cleaved‐caspase 3 and (D) Cyt *c*. Cyt *c*: cytochrome *c*. *P < .05 vs control group

Pre‐treatment with the PCSK9 inhibitor significantly decreased Bax expression, compared with the control group (Figure 4A). However, PCSK9 inhibitor given during ischaemia and at the onset of reperfusion did not alter Bax expression, compared with the control (Figure 4A). In addition, no significant difference in Bcl‐2 expression was observed in either of these treatment groups compared with the control group (Figure 4B). Our results also showed that the pre‐ischaemic administration of PCSK9 inhibitor significantly decreased cleaved‐caspase 3 levels when compared to the control group (Figure 4C). In terms of cytochrome *c*, only the PCSK9 inhibitor pre‐treatment group had decreased cytochrome *c* release levels when compared with the control group (Figure 4D).

### 3.6 PCSK9 inhibitor pre‐treatment decreased mitochondrial fission

**Figure 5:**
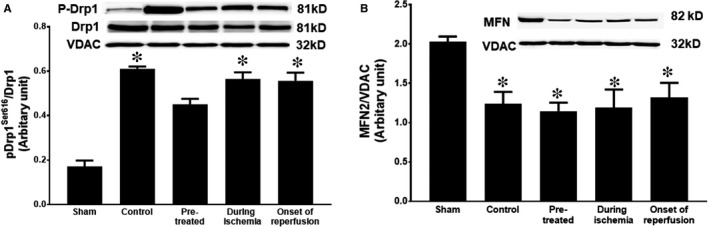
The effect of PCSK9 inhibitor on cardiac mitochondrial dynamics given at different time‐points (pre‐treatment, during ischaemia and at onset of reperfusion) during cardiac I/R. (A) Mitochondrial Drp1 level and (B) mitochondrial Mfn2 level. Drp1: dynamin‐related protein‐1; Mfn2: mitofusin; VDAC: voltage‐dependent anion channel. *P < .05 vs sham, †*P* < .05 vs control

The application of PCSK9 inhibitor and its potential effects on mitochondrial dynamics were investigated in terms of mitochondrial fusion and mitochondrial fission, regulated by mitofusin 2 (Mfn2) protein and dynamin‐related protein 1 (Drp1), respectively. Our results demonstrated a statistically significant decrease in the mitochondrial phosphorylated‐Drp1 at serine 616 in only the pre‐treated group when compared with the control group (Figure 5A). However, no significance in Mfn2 expression was observed between any treatment groups, compared with the control (Figure 5B).

### 3.7 PCSK9 inhibitor pre‐treatment improved left ventricular function

**Figure 6:**
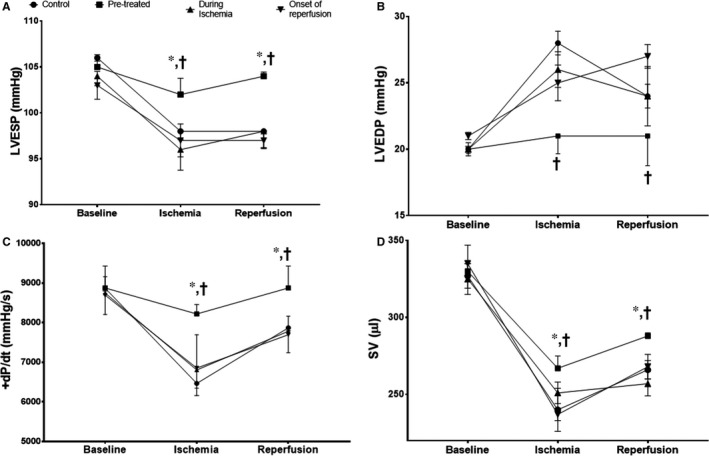
The effect of PCSK9 inhibitor on left ventricular function given at different time‐points (pre‐treatment, during ischaemia and at onset of reperfusion) during cardiac I/R. (A) Left ventricular end‐systolic pressure, (B) left ventricular end‐diastolic pressure, (C) +dP/dt max and (D) stroke volume. dP/dt: ventricular contractility assessment; LVEDP: left ventricular end‐diastolic pressure; LVESP: left ventricular end‐systolic pressure; SV: stroke volume. *P < .05 vs baseline of its group, †*P* < .05 vs control group at that period

To assess cardiac function, the left ventricular pressure‐volume loop relationships were measured. Our study demonstrated that only PCSK9 inhibitor pre‐treatment helped to improve the PV loop parameters (Figure 6A‐D)*.* There was no significant difference between the groups when compared to baseline values. During the ischaemic period, only pre‐treatment with the PCSK9 inhibitor improved left ventricular end‐systolic pressure (LVESP), left ventricular end‐diastolic pressure (LVEDP) and maximum + dP/dt and stroke volume when compared with the control group. At the reperfusion period, only LVESP and + dP/dt were improved by pre‐treatment with the PCSK9 inhibitor, when compared with the control group.

## 4 DISCUSSION

This investigation sought to determine the effects of PCSK9 inhibitor administered at several time‐points associated with cardiac I/R. Our study has shown that PCSK9 inhibitor treatment could be effective in attenuating biochemical and physiological complications seen in cardiac I/R injury. However, only pre‐ischaemia treatment with the PCSK9 inhibitor led to significant positive cardioprotective effects on cardiac function. PCSK9 inhibitor pre‐treatment was shown, relative to control, to result in the following: (a) improved mitochondrial function as indicated by a decrease in mitochondrial ROS, mitochondrial depolarization and mitochondrial swelling; (b) inhibition of mitochondrial fission; (c) attenuated cardiac arrhythmia via increased phosphorylation of Cx43; (d) decreased apoptosis related protein expression as indicated by decreased Bax, cytochrome *c* and cleaved‐caspase 3; (e) infarct size reduction; and (f) improved left ventricular function. Taken together, these data suggest that pre‐treatment with the PCSK9 inhibitor could convey cardioprotection during I/R injury.

Introduction of reperfusion therapy in post‐myocardial infarction treatment has increased the number of patients surviving the initial infarct; however, the proportion of patients developing heart failure has increased.^21^ This may in part be caused by the significant reperfusion injury sustained as blood flow is restored to the heart. A rapid increase in oxygen increases free radical synthesis in mitochondria to yield ROS, which subsequently cause many downstream cellular physiology effects, and leads to an overall change in cardiac function. Recently, it has been found, using in vitro techniques, that the use of PCSK9 is associated with a decrease in ROS generation.^14^, ^15^ In the present study, the inhibition of PCSK9 could have acted to attenuate mitochondrial damage by causing a decrease in ROS. Furthermore, pre‐treatment with the PCSK9 inhibitor was found to provide further mitochondrial protection by leading to reduced mitochondrial swelling and membrane potential depolarization. It is well established that ROS can influence the opening of the mitochondrial permeability pores (mPTP), causing loss of mitochondrial membrane potential depolarization resulting in an influx of water and solutes to increase swelling.^22^ Therefore, a decrease in mitochondrial ROS could be responsible for the decreased mitochondrial membrane depolarization and swelling found in the PCSK9 inhibitor pre‐treatment group.

In addition to the reduction in mitochondrial ROS, our results also clearly demonstrated that only the PCSK9 inhibitor pre‐treatment group had a lower arrhythmia score than the control group. Since oscillation of the mitochondrial membrane potential can cause fatal cardiac arrhythmia,^23^ one possible mechanism could be through its ability to reduce cardiac mitochondrial membrane depolarization caused by I/R injury. In addition, the anti‐arrhythmic effects of the PCSK9 inhibitor could be because of its effect on the Cx43. Cx43 is a cardiac gap junction protein, which facilitates cardiomyocyte communication via electrical current flow.^24^ Phosphorylation of Cx43 at serine 368 increased the trafficking of Cx43 to the plasma membrane, leading to the generation of a gap junction.^24^ In this study, pCx43 was decreased in cardiac I/R, whereas it was increased in the PCSK9 inhibitor pre‐treated group.

It is also known that excessive mitochondrial ROS production during the period of I/R results in an impairment of mitochondrial dynamic processes via excessive fission and fragmentation, which is associated with cardiac cell apoptosis.^25^, ^26^, ^27^, ^28^ During the period of I/R, our results clearly demonstrated an increase in mitochondrial dynamic imbalance as indicated by increased phosphorylation of Drp1. Moreover, excessive mitochondrial fission, concomitant with Bax activation and cytochrome c release, activated caspase and led to cardiomyocyte apoptosis. In the present study, pre‐treatment with the PCSK9 inhibitor was found to decrease mitochondrial Drp1 and apoptotic‐associated proteins. In addition, a previous study demonstrated that pre‐treatment with anti‐PCSK9 siRNA inhibited the caspase 9‐caspase 3 pathway via an increase in Bcl‐2 and a decline in Bax.^29^ All of these points could explain our finding that pre‐treatment with the PCSK9 inhibitor led to significantly decreased Drp1 expression, which resulted in reduced Cyt c, and cleaved‐caspase 3 expression.

**Figure 7:**
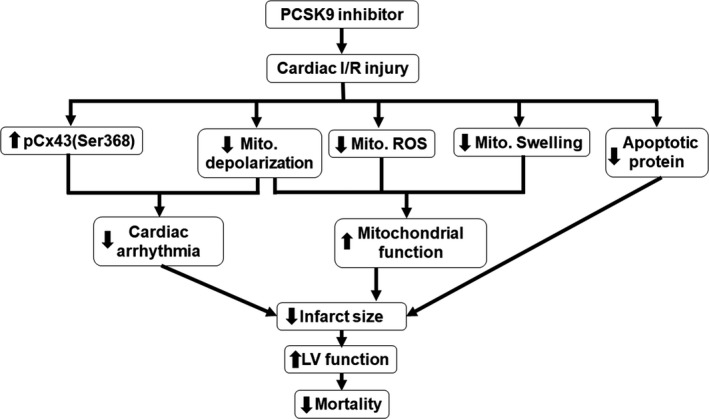
A summary of the beneficial effects of pre‐treatment with PCSK9 inhibitor during cardiac I/R as observed in this study

All cardiac I/R injury studies have been mainly focusing on a reduction in infarct size.^30^, ^31^, ^32^ Our study demonstrated that only pre‐treatment with the PCSK9 inhibitor provided cardioprotective effects demonstrated by a markedly reduced infarct size. The underlying mechanism for the reduction in the infarct size by the PCSK9 inhibitor could be because of its ability to attenuate cardiac mitochondrial dysfunction and mitochondrial fission and decrease the apoptotic process in the ischaemic myocardium. All of these beneficial effects could be responsible for the improved LV function observed in this study as shown in Figure 7. In addition, the underlying mechanism responsible for the cardioprotection found in the pre‐treatment group could be because of the timing of PCSK9 inhibition. It has been shown that the sustained PCSK9 release during cardiac I/R could lead to cardiac deleterious effects by inducing cell death and dysfunction.^15^ In addition, previous studies found that PCSK9 inhibitor causes a decrease in the level of oxidative stress by increased Cu, Zn‐superoxide dismutase (SOD), catalase and C‐reactive protein in patients with coronary artery disease.^33^, ^34^ Therefore, PCSK9 inhibitor given prior to ischaemic period might be a prophylactic approach to prevent cardiac adverse effects after I/R injury as we found in this study. In the present study, PCSK9 inhibitor given after ischaemic period did not provide cardioprotective effects. This could be because of the fact that PCSK9 was already released and bound with its receptors during myocardial ischaemia. Therefore, PCSK9 inhibitor given after myocardial ischaemia could not prevent their binding, thus no benefits as cardioprotection was observed. However, whether the higher concentration of PCSK9 inhibitor given during ischaemia or after reperfusion could provide these benefits is not known. Future studies are needed to investigate whether the beneficial effects of this PCSK9 inhibitor is dose‐dependent and whether the protection can be obtained if a higher concentration of PCSK9 inhibitor is used when it is given during ischaemia and/or at the onset of reperfusion.

In conclusions, the PCSK9 inhibitor given only prior to ischaemia exerts cardioprotection against the injury triggered by I/R. The cardioprotective mechanisms included an increase in connexin43 phosphorylation, improved cardiac mitochondrial function, and a decrease in apoptosis, leading to attenuated cardiac arrhythmia and a reduction in infarct size, ultimately resulting in an improvement in left ventricular function. These findings have highlighted the potential use of this novel PCSK9 inhibitor outside of hyperlipidemia and atheroprotection, thus providing significant impetus for the need for future clinical investigations.

## ACKNOWLEDGEMENTS

This work was supported by Thailand Research Fund grants: RSA6180056 (SP), RTA6080003 (SCC), TRF‐Royal Golden Jubilee Program (CM and NC) and, the NSTDA Research Chair grant from the National Science and Technology Development Agency Thailand (NC), and the Chiang Mai University Center of Excellence Award (NC).

## CONFLICT OF INTEREST

The authors declare that they have no conflict of interest.

## AUTHOR CONTRIBUTIONS

SP, SCC and NC designed the experiments and wrote the paper. SP, CMM, DMM, CM, TJ and SK performed the experiments. SP, CMM and CM analysed the data. All authors approved the final version of the paper.

## Data Availability

All data are presented in the results of this manuscript.

## References

[1] WHO .Mortality and global health estimates. 2017.

[2] Rizzo M , Rini GB , Berneis K . Effects of statins, fibrates, rosuvastatin, and ezetimibe beyond cholesterol: the modulation of LDL size and subclasses in high‐risk patients. Adv Ther. 2007;24:575‐582. 17660166 10.1007/BF02848780

[3] Cainzos‐Achirica M , Martin SS , Cornell JE , Mulrow CD , Guallar E . Pcsk9 inhibitors: a new era in lipid‐lowering treatment? Ann Intern Med. 2015;163:64‐65. 25915768 10.7326/M15-0920

[4] Wu N‐Q , Li S , Li J‐J . Update of clinical trials of anti‐PCSK9 antibodies. Cardiovasc Drug Ther. 2015;29:159‐169. 10.1007/s10557-015-6582-9 25916406

[5] Horton JD , Cohen JC , Hobbs HH . PCSK9: a convertase that coordinates LDL catabolism. J Lipid Res. 2009;50:S172‐S177. 19020338 10.1194/jlr.R800091-JLR200 PMC2674748

[6] Grefhorst A , McNutt MC , Lagace TA , Horton JD . Plasma PCSK9 preferentially reduces liver LDL receptors in mice. J Lipid Res. 2008;49:1303‐1311. 18354138 10.1194/jlr.M800027-JLR200 PMC2386900

[7] Liu ZP , Wang Y . PCSK9 Inhibitors: novel therapeutic strategies for lowering LDL‐cholesterol. Mini Rev Med Chem. 2018;19(2):165‐176. 10.2174/1389557518666180423111442 29692249

[8] Ferdinandy P . Myocardial ischaemia/reperfusion injury and preconditioning: effects of hypercholesterolaemia/hyperlipidaemia. Br J Pharmacol. 2009;138:283‐285. 10.1038/sj.bjp.0705097 PMC1573675 12540517

[9] Tompkins AJ , Burwell LS , Digerness SB , Zaragoza C , Holman WL , Brookes PS . Mitochondrial dysfunction in cardiac ischemia–reperfusion injury: ROS from complex I, without inhibition. Biochim Biophys Acta. 2006;1762:223‐231. 16278076 10.1016/j.bbadis.2005.10.001

[10] Cadenas S . ROS and redox signaling in myocardial ischemia‐reperfusion injury and cardioprotection. Free Radic Biol Med. 2018;117:76‐89. 29373843 10.1016/j.freeradbiomed.2018.01.024

[11] von Harsdorf R , Li PF , Dietz R . Signaling pathways in reactive oxygen species‐induced cardiomyocyte apoptosis. Circulation. 1999;99:2934‐2941. 10359739 10.1161/01.cir.99.22.2934

[12] Zhao Z‐Q , Velez DA , Wang N‐P , et al. Progressively developed myocardial apoptotic cell death during late phase of reperfusion. Apoptosis. 2001;6:279‐290. 11445670 10.1023/a:1011335525219

[13] Stone GW , Selker HP , Thiele H , et al. Relationship between infarct size and outcomes following primary PCI. J Am Coll Cardiol. 2016;67:1674‐1683. 27056772 10.1016/j.jacc.2016.01.069

[14] Ding Z , Liu S , Wang X , et al. Cross‐talk between LOX‐1 and PCSK9 in vascular tissues. Cardiovasc Res. 2015;107:556‐567. 26092101 10.1093/cvr/cvv178

[15] Ding Z , Wang X , Liu S , et al. PCSK9 expression in the ischemic heart and its relationship to infarct size, cardiac function and development of autophagy. Cardiovasc Res. 2018;114(13):1738‐1751. 29800228 10.1093/cvr/cvy128

[16] Curtis MJ , Hancox JC , Farkas A , et al. The lambeth conventions (II): guidelines for the study of animal and human ventricular and supraventricular arrhythmias. Pharmacol Ther. 2013;139(2):213‐248. 23588158 10.1016/j.pharmthera.2013.04.008

[17] Kroekkiat C , Jantira S , Siriporn C , Nipon C . Dipeptidyl peptidase‐4 inhibitor reduces infarct size and preserves cardiac function via mitochondrial protection in ischaemia–reperfusion rat heart. Diab Vasc Dis Res. 2013;11:75‐83. 24357666 10.1177/1479164113516134

[18] Riess ML , Rhodes SS , Stowe DF , Aldakkak M , Camara A . Comparison of cumulative planimetry versus manual dissection to assess experimental infarct size in isolated hearts. J Pharmacol Toxicol Methods. 2009;60:275‐280. 19732842 10.1016/j.vascn.2009.05.012 PMC3786703

[19] Apaijai N , Chinda K , Palee S , Chattipakorn S , Chattipakorn N . Combined vildagliptin and metformin exert better cardioprotection than monotherapy against ischemia‐reperfusion injury in obese‐insulin resistant rats. PLoS ONE. 2014;9:e102374. 25036861 10.1371/journal.pone.0102374 PMC4103813

[20] Thummasorn S , Apaijai N , Kerdphoo S , Shinlapawittayatorn K , Chattipakorn SC , Chattipakorn N . Humanin exerts cardioprotection against cardiac ischemia/reperfusion injury through attenuation of mitochondrial dysfunction. Cardiovasc Ther. 2016;34:404‐414. 27434747 10.1111/1755-5922.12210

[21] Cahill TJ , Kharbanda RK . Heart failure after myocardial infarction in the era of primary percutaneous coronary intervention: mechanisms, incidence and identification of patients at risk. World J Cardiol. 2017;9:407‐415. 28603587 10.4330/wjc.v9.i5.407 PMC5442408

[22] Makazan Z , Saini HK , Dhalla NS . Role of oxidative stress in alterations of mitochondrial function in ischemic‐reperfused hearts. Am J Physiol Heart Circ Physiol. 2007;292:H1986‐H1994. 17172267 10.1152/ajpheart.01214.2006

[23] Aon MA , Cortassa S , O'Rourke B . Mitochondrial oscillations in physiology and pathophysiology. Adv Exp Med Biol. 2008;641:98‐117. 18783175 10.1007/978-0-387-09794-7_8 PMC2692514

[24] Lampe PD , TenBroek EM , Burt JM , Kurata WE , Johnson RG , Lau AF . Phosphorylation of connexin43 on serine368 by protein kinase C regulates gap junctional communication. J Cell Biol. 2000;149:1503‐1512. 10871288 10.1083/jcb.149.7.1503 PMC2175134

[25] Ong SB , Subrayan S , Lim SY , Yellon DM , Davidson SM , Hausenloy DJ . Inhibiting mitochondrial fission protects the heart against ischemia/reperfusion injury. Circulation. 2010;121:2012‐2022. 20421521 10.1161/CIRCULATIONAHA.109.906610

[26] Cui M , Ding H , Chen F , Zhao Y , Yang Q , Dong Q . Mdivi‐1 protects against ischemic brain injury via elevating extracellular adenosine in a cAMP/CREB‐CD39‐dependent manner. Mol Neurobiol. 2016;53:240‐253. 25428621 10.1007/s12035-014-9002-4

[27] Disatnik MH , Ferreira JC , Campos JC , et al. Acute inhibition of excessive mitochondrial fission after myocardial infarction prevents long‐term cardiac dysfunction. J Am Heart Assoc. 2013;2:e000461. 24103571 10.1161/JAHA.113.000461 PMC3835263

[28] Sharp WW , Fang YH , Han M , et al. Dynamin‐related protein 1 (Drp1)‐mediated diastolic dysfunction in myocardial ischemia‐reperfusion injury: therapeutic benefits of Drp1 inhibition to reduce mitochondrial fission. FASEB J. 2014;28:316‐326. 24076965 10.1096/fj.12-226225 PMC3868827

[29] Dewson G , Kluck RM . Mechanisms by which Bak and Bax permeabilise mitochondria during apoptosis. J Cell Sci. 2009;122:2801‐2808. 19795525 10.1242/jcs.038166 PMC2736138

[30] Zhu J , Su X , Li G , Chen J , Tang B , Yang Y . The incidence of acute myocardial infarction in relation to overweight and obesity: a meta‐analysis. Arch Med Sci. 2014;10:855‐862. 25395935 10.5114/aoms.2014.46206 PMC4223131

[31] Hausenloy DJ , Yellon DM . Myocardial ischemia‐reperfusion injury: a neglected therapeutic target. J Clin Invest. 2013;123:92‐100. 23281415 10.1172/JCI62874 PMC3533275

[32] Yellon DM , Hausenloy DJ . Myocardial reperfusion injury. N Engl J Med. 2007;357:1121‐1135. 17855673 10.1056/NEJMra071667

[33] Lankin VZ , Tikhaze AK , Viigimaa M , Chazova IЕ . PCSK9 Inhibitor causes a decrease in the level of oxidatively modified low‐density lipoproteins in patients with coronary artery diseases. Ter Arkh. 2018;90:27‐30. 10.26442/terarkh201890927-30 30701731

[34] Tobaru T , Seki A , Asano R , Sumiyoshi T , Hagiwara N . Lipid‐lowering and anti‐inflammatory effect of ezetimibe in hyperlipidemic patients with coronary artery disease. Heart Vessels. 2013;28:39‐45. 22427252 10.1007/s00380-012-0243-8

